# Level of CD8 T Lymphocytes Activation in HIV-Infected Pregnant Women: In the Context of CD38 and HLA-DR Activation Markers

**DOI:** 10.1155/2014/715279

**Published:** 2014-01-22

**Authors:** Stanslaus Musyoki, Simeon Mining, Paul Nyongesa

**Affiliations:** ^1^Kisii University, P.O. Box 408-40200, Kisii, Kenya; ^2^Department of Immunology, Moi University, P.O. Box 4606-30100, Eldoret, Kenya; ^3^Department of Reproductive Health, Moi University, P.O. Box 4606-30100, Eldoret, Kenya

## Abstract

*Background*. To date the effect of pregnancy on the immune activation of CD8 T cells that may affect HIV disease progression has not been well studied and remains unclear. *Objective.* To determine the effect of pregnancy on CD8 T lymphocyte activation and its relationship with CD4 count in HIV infected pregnant women. *Study Design*. Case control. *Study Site*. AMPATH and MTRH in Eldoret, Kenya. *Study Subjects*. Newly diagnosed asymptomatic HIV positive pregnant and nonpregnant women with no prior receipt of antiretroviral medications. *Study Methods*. Blood samples were collected from the study participants and levels of activated CD8 T lymphocytes (CD38 and HLA-DR) were determined using flow cytometer and correlated with CD4 counts of the study participants. The descriptive data focusing on frequencies, correlation, and cross-tabulations was statistically determined. Significance of the results was set at *P* < 0.05. *Results*. HIV positive pregnant women had lower activated CD8 T lymphocyte counts than nonpregnant HIV positive women. Activated CD8 T lymphocyte counts were also noted to decrease in the second and third trimesters of pregnancy. *Conclusion*. Pregnancy has a significant suppression on CD8+ T lymphocyte immune activation during HIV infections. Follow-up studies with more control arms could confirm the present study results.

## 1. Introduction

Cytotoxic T lymphocyte (CD8) cell activation is a major cause of HIV pathology [1]. The expression of the activation markers CD38 and HLA-DR on T cells is an indicator of cell activation [2]. HIV infection activates CD8 T cells resulting in their expansion and expression of HLA-DR antigens [3]. To date the effect of pregnancy on the immune activation of CD8 T cells that may affect HIV disease progression has not been well studied and remains unclear. Thus, the study set to investigate the levels of activated CD8^+^ T surface markers (CD38 and HLA-DR) would lead to a better understanding of the immunological relationship of HIV and pregnancy. The objective of the study was therefore to compare CD8 T lymphocyte activation surface markers (CD38 and HLA-DR) levels in pregnant and nonpregnant women with HIV infection. The study used CD8 cellular immune activation surface markers (CD8^+^/HLA-DR/CD38) because CD8^+^ cellular activation, as opposed to CD4^+^ activation, is more predictive of long-term immunologic responses as CD4^+^ cells are infected by HIV and are more likely to be removed through apoptosis [4].

## 2. Materials and Methods

The investigation was done in Eldoret, Kenya, at Academic Model for Providing Access to Healthcare (AMPATH) centre outpatient clinics and Moi Teaching and Referral Hospital (MTRH)—Mother Child Health Care (MCH) Clinics. AMPATH was started in 2000 as part of collaboration between Moi University (MU), MTRH, and a consortium of American universities led by Indiana University of the United States. AMPATH is the largest HIV/AIDS treatment and care program in Kenya. MTRH is the second largest hospital in Kenya and serves referral patients from Rift Valley and Western Kenya. AMPATH aims at a comprehensive care for HIV-infected patients, enhancing education and research in HIV/AIDS.

In a case-controlled study design asymptomatic HIV-positive pregnant and nonpregnant women with no prior receipt of antiretroviral medications and ages ≥18 years and ≤40 years were evaluated. A study sample guideline-questionnaire was used to enquire from the study participants the possible period of HIV infection with ≥6 months set as the maximum period of HIV infection to recruit the study participants. This was done for both study group and controls since it is naturally difficult to recruit participants of exactly similar time after infection; this was one of the study limitations. Women with a history or diagnosis of any complications in pregnancy (previous or present), for example, miscarriages, tumors, diabetic mellitus, pneumonia, influenza, herpes, autoimmunity, or evidence of acute infection, and untreated medical illness, for example, coinfections in pregnancy, were excluded from the study. Consecutive sampling technique was used to recruit thirty-six HIV-infected pregnant women as the study subjects and thirty-six HIV-infected nonpregnant women as control group. Specific levels of CD8^+^ cellular immune activation markers (CD8^+^/HLA-DR/CD38) were determined and correlated with CD4 counts between the two groups.

Laboratory analysis to determine the levels of activated CD8 T lymphocytes was performed using a flow-cytometer FACSCalibur (BD Biosciences, San Jose, CA, USA). The flow-cytometer machine was equipped with 635 nm and 488 nm lasers and could detect four color fluorescence with emissions detectable in the ranges of 515–545 nm, 562–607 nm, >650 nm, and 652–608 nm. The machine also set to discriminate using >650 channel. Blood samples were stained within 48 hours of collection and analyzed within 24 hours of staining. CD8^+^/HLA-DR/CD38 surface markers were then labeled and analysed using CD38 and HLA-DR monoclonal antibodies conjugated with FITC: fluorescein isothiocyanate; PE: phycoerythrin; PE-C5: phycoerythrin-cyanine5; APC: allophycocyanin. processing of the samples was done using FACS Lyse/No wash method, acquired from a BD FACSCalibur instrument. For each sample mixed with anticoagulant EDTA, tubes were prepared. Twenty (20) *μ*L of the antibodies were put in each tube and vortexed. Then 50 *μ*L of the blood sample was added, vortexed, and incubated for 15 minutes. After staining and following incubation, cells were lysed with 450 *μ*L of BDFacLyse reagent and incubated for at least another 15 minutes. Live gating based on forward- and side-scatter properties was set on the CD3^+^ and CD8^+^ lymphocyte fraction. Live gating was used to collect 10 000 events by FSC and SSC cytogram with a gate set on the CD3^+^ and CD8^+^ lymphocyte fraction. The cells were gated based on forward- and side-scatter properties. The percentage of cells CD8^+^ T cells expressing CD38 and HLA-DR surface markers was then read using the cell quest pro software (BD Biosciences, San Jose, CA, USA). All the data was recorded on a standard case report forms, a tool developed for this study. Statistical analysis of descriptive data focusing on frequencies, correlation, and cross-tabulations was determined using statistical package for social sciences (SPSS) version 16.0 (Norusis, SPSS, Chicago, IL, USA). Non-parametric methods were used to statistically evaluate the data and compare tests since the data was not normally distributed. Significance of the results was set at *P* < 0.05.

The study was given ethical approval by Institutional Research and Ethics Committee (IREC) from Moi University/Moi Teaching and Referral Hospital (approval number is 000388). The study procedures did not interfere in any manner with the routine clinical care provided to patients enrolled in the study. Informed and voluntary consent by each subject was sought before any data was collected from the subjects. The purpose, procedures, and risks and benefits of the study were also discussed with the subject. The participants were given adequate opportunities to discuss and contemplate their participation. The participants also retained the right to refuse to answer individual questions or to discontinue study participation without jeopardy. The participants had privacy and all information was treated as confidential.

## 3. Results

Social-economic and demographic characteristics have been shown in Table 1. The mean age of the 72 women included in this analysis at the time that it was performed was 30.35 years with that of the 36 nonpregnant women being 30.75 years and pregnant ones 29.94 years. 52 (72.2%) were married and 56 (77.8%) were unemployed or earned less than KSH10, 000 (125 US dollars) per month as income. No statistically significant differences were found in the distribution of age, marital status, and income (*P* > 0.299) between the two study groups and in subjects of different trimesters of pregnancy. Statistical difference was however observed in the CD4 absolute count.

The study showed marked and significant lower activated CD8 T lymphocyte surface markers in pregnant women compared to the nonpregnant group (CD8/HLA-DR % (*P* = 0.029) and CD8/CD38 % (*P* = 0.011)) as depicted in Table 2. Medians found in the CD8^+^ cells expressing HLA-DR and CD38 in nonpregnant HIV-infected individuals were 31.6% and 32.0%, respectively, while in the HIV pregnant women they were 28.4% and 27.3%, respectively.

Variation of CD8^+^ cellular immune activation markers in the pregnant group in different trimesters is as shown in Table 3. The CD8^+^ T cell immune activation markers (CD8/HLA-DR/CD38) varied significantly in the different trimesters of pregnancy (*P* < 0.028). The highest percent counts were observed in first trimester with a decrease towards the third trimester. The medians of percent CD8^+^ immune activation marker CD8/HLA-DR in the first, second, and third trimesters were 39.2%, 28.4%, and 25.3%, respectively. Similarly the CD8/CD38 median percent counts in the subjects in the first, second, and third trimesters were 39.3%, 28.4%, 24.8%, respectively.

The relationship between the activated CD8^+^ T cells (CD8/HLA-DR/CD38) and CD4^+^ cell counts have been shown in Tables 4 and 5. The expression of CD8^+^/HLA-DR (denoted by A) by CD8^+^ T cells was correlated with CD8/CD38 and CD4^+^ cells (all denoted by B). When CD8^+^ immune activation markers were correlated in both study groups it was observed that the surface marker CD8^+^/HLA-DR positively correlated at 99% confidence level with CD8^+^/CD38 (*P* < 0.001, *r* = 0.999) indicating that when high percent counts of CD8^+^/HLA-DR are observed, high percent CD8^+^/CD38 counts were also noted in the study subjects. The percentage of CD8^+^ T cells that were CD38^+^ and HLA-DR^+^ had inverse correlations with both the percentage and absolute counts of CD4^+^ T cells in both study groups. However, nonsignificant inverse correlation for the CD8^+^ cellular immune activation markers (HLA-DR and CD38) and CD4^+^ cell counts was observed in the pregnant study group. In the nonpregnant group CD4^+^, absolute cell counts showed a 99% confidence level of negative correlation with the two surface markers of CD8^+^ immune activation (HLA-DR and CD38). CD4^+^ percent cell counts correlated negatively and significantly at 95% confidence level with the CD8^+^ cellular immune activation markers (HLA-DR and CD38) in the nonpregnant subjects.

## 4. Discussion and Conclusions

The present study investigated how pregnancy alters CD8 T cell immune activation and how this may correlate with CD4 counts, as an indicator of immune status. No statistically significant differences were found in the demographics and social economic characteristics between the two groups as shown in Table 1. Pregnancy appears to depress expression of activated CD8 T lymphocytes. It has been shown that the total CD8^+^ lymphocytes may not be altered by pregnancy [5], but the present study indicates decrease in percentage of activated CD8^+^ T cells in HIV-infected pregnant women. Progressive suppression was also observed in different trimesters of pregnancy in HIV-infected women as shown in Table 3. This provides evidence that nonpregnant women can expect high turnover and effector function of activated CD8^+^ T cells than pregnancy with HIV infection. The data presented herein support the contention that high levels of CD38 and HLA-DR expression correlate with CD8^+^ T cell subset activation in HIV^+^ individuals [3, 6]. Chronic immune activation and constant turnover of activated CD8^+^ T cells are therefore a hallmark of HIV infection, and these factors are now thought of as playing a critical role in HIV pathogenesis and disease progression by increased rate of CD4^+^ T cell depletion [7]. CD38 and HLA-DR expression on CD8^+^ T cell subsets correlated inversely (significantly in nonpregnant subjects) with CD4^+^ T cell counts as shown in Tables 4 and 5. This observation suggests that expression of these markers is an important marker for CD8^+^ T cell activation and CD4^+^ cell depletion, particularly in HIV infection with no pregnancy. Immune activation-driven death of CD4^+^ cells, more than a direct virological pathogenic effect, could be responsible for decline in CD4^+^ T cells [7, 8]. Thus, HLA-DR and CD38 expression on CD8^+^ T lymphocytes may serve as a tag to identify those CD4^+^ T cells that are being eliminated by activation-induced killing during HIV infection. Based on the study results CD8^+^ CD38^+^ DR^+^ T cell level seems to be depressed in pregnant HIV-infected patients and therefore may be an important prognostic parameter of immune status during pregnancy. The present study design could not show the cause-effect relationship but gave a mere comparison of study groups and relationships of variables; this could be rigorously studied using follow-up study design with more control arms including viral load counts to validate the observed results.

## Figures and Tables

**Table 1:** Social-economic and demographic characteristics of the study groups.

Characteristics	Pregnant	Nonpregnant	*P* value	Total
Mean age	29.94 (SD = 4.91)	30.75 (SD = 4.62)	0.587	30.35 (SD = 4.75)
Income				
Less than 125 US dollars per month	27 (75%)	29 (80.6%)	0.577	56 (77.8%)
Equal or more than 125 US dollars per month	9 (25%)	7 (19.40%)		16 (22.2%)
Marital status				
Married	28 (77.8%)	24 (66.7%)	0.299	52 (72.2%)
Not married	8 (22.2%)	12 (33.3%)		20 (27.8%)
Absolute CD4 count (median (IQR))	442.5 (331.5, 539.5)	316 (178.5, 485)	0.004	
WHO staging	Stage I	Stage I		

**Table 2:** Activated CD8T cell percent counts in the study groups (data are median (interquartile range)).

Parameters	Pregnant women	Nonpregnant women	*P* values
CD8/HLADR %	28.43 (23.47, 37.49)	31.64 (29.25, 36.44)	0.029*
CD8/CD38 %	27.29 (23.19, 36.41)	31.9600 (29.0925, 36.4775)	0.011*

*Significant *P* values.

**Table 3:** Activated CD8T cell counts in the pregnant group in different trimesters (data are median (interquartile range)).

Parameters	Trimesters			*P* values
	1st (<10 wks) (*n* = 5)	2nd (10–26 wks) (*n* = 14)	3rd (>26 wks) (*n* = 17)	
CD8/HLADR %	39.2 (32.7, 44.5)	28.4 (25.7, 34.9)	25.3 (22.5, 38.3)	0.028*
CD8/CD38 %	39.3 (33, 44.6)	28.4 (25.8, 34.9)	24.8 (19.6, 32.3)	0.008*

*Significant *P* values

**Table 4:** Relationship between CD8/HLA-DR, CD8/38, and the CD4^+^ cell counts (statistical analysis by Pearson correlation (Sig. (2 tailed))).

CD8/HLA-DR % (A)	Counts (B)	Nonpregnant		Pregnant	
		Correlation coefficient (*r*)	*P* value	Correlation coefficient (*r*)	*P* values
	CD8/38 %	*r* = 0.999**	<0.001	*r* = 0.876**	<0.001
	Absolute CD4	*r* = −0.470**	0.004	*r* = −0.056	0.746
	CD4 %	*r* = −0.153	0.374	*r* = −0.022	0.898

A: CD8^+^/HLA-DR cellular immune activation markers; B: CD8/CD38 and CD4 cell counts.

**Correlation is significant at the 0.01 level (2 tailed).

**Table 5:** Relationship between CD8/38 and the CD4^+^ cell counts (statistical analysis by Pearson correlation (Sig. (2 tailed))).

CD8/38 % (A)	Counts (B)	Nonpregnant		Pregnant	
		Correlation coefficient (*r*)	*P* value	Correlation coefficient (*r*)	*P* values
	Absolute CD4	*r* = −0.478**	0.003	*r* = −0.277	0.102
	CD4 %	*r* = −0.154	0.370	*r* = −0.162	0.345

A: CD8/38 cellular immune activation markers; B: CD4 cell counts.

**Correlation is significant at the 0.01 level (2 tailed).

*Correlation is significant at the 0.05 level (2 tailed).
